# Unveiling basidiobolomycosis: key imaging features and clinical correlations

**DOI:** 10.1186/s12879-025-11781-x

**Published:** 2025-10-21

**Authors:** Fatemeh Yarmahmoodi, Mohammadreza Sheikhfendereski, Seyed Mostajab Razavinejad, Banafsheh Zeinali-Rafsanjani, Mahdi Saeedi-Moghadam

**Affiliations:** 1 Medical Imaging Research Center, Shiraz University of Medical Sciences, Shiraz, Iran; 2 Department of Radiology, Shiraz University of Medical Sciences, Shiraz, Iran; 3 Neonatal Research Center, Shiraz University of Medical Sciences, Shiraz, Iran

**Keywords:** Basidiobolomycosis, Fungal infection, Radiologic findings, Prognosis

## Abstract

**Background:**

Basidiobolomycosis is a fungal infection exhibiting a wide spectrum of clinical manifestations that frequently resemble abdominal malignancies or inflammatory conditions. This study elucidates the characteristic imaging features that can help make an accurate diagnosis.

**Methods:**

We examined pretreatment imaging studies (CT, MRI, ultrasound) of 32 histopathologically proven cases of basidiobolomycosis at a tertiary referral center between 2015 and 2021. Two radiologists, blind to the pathology results, scrutinized the lesions for localization, morphology, enhancement pattern, and ancillary findings.

**Results:**

The cohort (mean age 9.8 ± 13.8 years and 53% male) demonstrated three basic imaging patterns: (1) hepatic lesions (21.9%) appearing as heterogeneously enhancing masses with necrotic cores; (2) gastrointestinal involvement (18.8%) showing circumferential wall thickening chiefly affecting the ileocecal region; and (3) mesenteric masses (15.6%) with peripheral enhancement and central necrosis. Other findings included regional lymphadenopathy (31.3%) and obstructive uropathy. At first, the misdiagnosis rate was 78%, most frequently as malignancy (37.5%) or appendicitis (31.2%). The follow-up indicated that 90.6% were responsive to treatment.

**Conclusion:**

Basidiobolomycosis shows distinct imaging characteristics, especially necrotic hepatic masses, and segmental bowel wall thickening, which could help differentiate the condition from neoplasms or inflammatory diseases. These findings act as a compelling argument to always include fungal etiologies within the differential diagnosis in patients with pertinent imaging findings in endemic regions.

## Introduction

Basidiobolomycosis, which has been caused by an environmental fungus called Basidiobolus ranarum, represents an emerging diagnostic challenge to tropical medicine. While first documented in 1956 as cutaneous infection, the gastrointestinal (GI) form has shown an increase in recognition as an organized entity accounting for 15–20% of cases found in endemic regions [1, 2].

The GI variant is of special clinical importance due to its aggressive presentation and common misdiagnosis. Molecular studies confirm food- or soil-borne transmission from ingested spores, while outbreaks from contaminated agricultural products have been documented [3]. This pathogenesis explains why it mostly occurs in arid regions, like southern Iran, Saudi Arabia, and Arizona, where the per capita prevalence is 0.8 per 100,000-near what some bacterial enteropathies would show [4, 5].

Diagnostic challenges include multifactorial originated sources: 1) Clinical mimicry: 68–78% of cases misdiagnosed as malignancy (37–45%) or IBD (18–22%) [6, 7]; 2) Temporal delays in procedural: Time-to-diagnosis = 6.2 weeks on average in Iran [8]. 3) Invasive need: The current gold standard requires a biopsy to demonstrate the Splendore-Hoeppli phenomenon [3]. 4) These delays in diagnosis cause obvious harm: 22% of patients in one Saudi series had unnecessary cancer resections before accurate diagnosis [9], studies from southern Iran report 6.2-week delays in treatment commencement [5].

Radiology still has potential not fully exploited despite advances in technology. While retrospective analysis indicates that bowel wall thickening occurs (72–85%) and hepatic masses (15–30%) [10]. no studies have systematically analyzed distinguishing imaging features [11]. The critical gaps remain with no standardized imaging criteria, poor characterization of enhancement patterns, and scanty data on MRI specificity.

This gap is rather in stark contrast to other fungal infections, where imaging is the defining tool, e.g., in hepatosplenic candidiasis by “bull’s-eye” lesions [12]. This problem is acute in southern Iran because basidiobolomycosis accounts for 12% of pediatric abdominal mass referrals at University-affiliated hospitals [13]. Regional studies highlight basidiobolomycosis as a developing cause of infectious colitis in immunocompetent children [3].

This study is therefore organized to characterize systematically the CT/MRI features, develop evidence-based diagnostic criteria, and differentiate basidiobolomycosis from malignancy/IBD. These unresolved challenges require systematic solutions, especially in endemic regions such as southern Iran. Also, this study is in line with the WHO priority pathogen list related to fungal diseases which puts emphasis on ‘developing rapid diagnostics for neglected fungal pathogens’ [14]. To do so, the radiological findings of patients with a definite diagnosis of basidioblomycosis in the University-affiliated hospital, during the years of 2015–2021 were investigated.

## Material and method

All pathology-proven basidioblomycosis patients who had at least one medical imaging before surgical and medical intervention after admission to the hospital from February 1, 2014, to March 29, 2021 were included in the study. Patients who had received any surgical or medical intervention before imaging, immunocompromised individuals, e.g., HIV patients or chemotherapy or transplant recipients, patients who had not had histopathological confirmation of their disease, and patients with incomplete imaging records were excluded from the study.

In order to collect information, at first, the patients’ names were extracted from the hospital archives. Their files were evaluated regarding demographic characteristics (age and gender), history, clinical examinations, laboratory findings, and treatment outcomes. Two expert radiologists, having spent 15 and 12 years working in the field respectively, conducted all analyses on the imaging studies independently. Even if interobserver statistics had not formally been conducted owing to the retrospective design of the experiment and the need to get consensus clinically, it suffices to mention that disagreement (*n* = 3 cases) was settled through joint re-evaluation and discussion with a third senior radiologist (25 years’ experience).

**Fig. 1:**
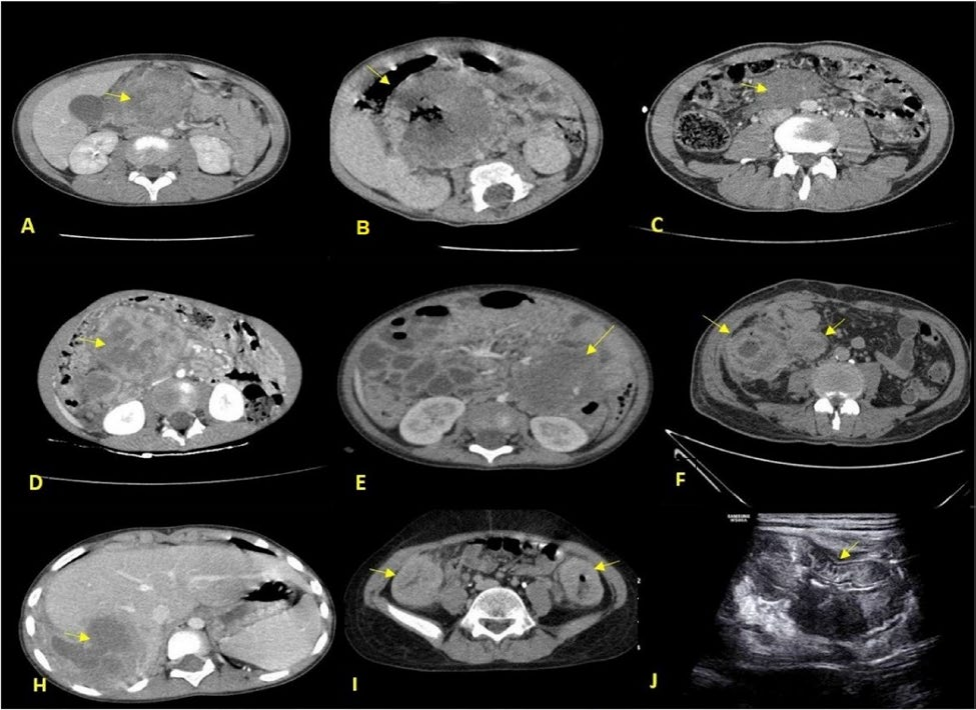
(**A**) A large mass of the mesentery in the epigastrium with peripheral heterogeneous enhancement and central necrosis with involvement of the head of pancreas and vascular encasement, who underwent Whipple surgery. (**B**) A large mesenteric mass in the right upper quadrant of the abdomen with peripheral heterogeneous enhancement and central necrosis. (**C**) A mesenteric mass in the lower abdomen with heterogeneous enhancement. (**D**) A mesenteric mass in the right side of the abdomen with heterogeneous enhancement and central necrosis. (**E**) A mesenteric mass in the left side of the abdomen with heterogeneous enhancement and central necrosis also shows an increase in the thickness of the adjacent colon. (**F**) Circumferential wall thickening of cecum and terminal ileum. **H**) Hepatic mass with heterogeneous enhancement and central necrosis in the right lobe of the liver with extension to the perihepatic space. **I**) Increasing the thickness of the ascending and descending colon with heterogeneous enhancement. **J**) Ultrasound view of the increase in the thickness of the ascending colon

**Fig. 2:**
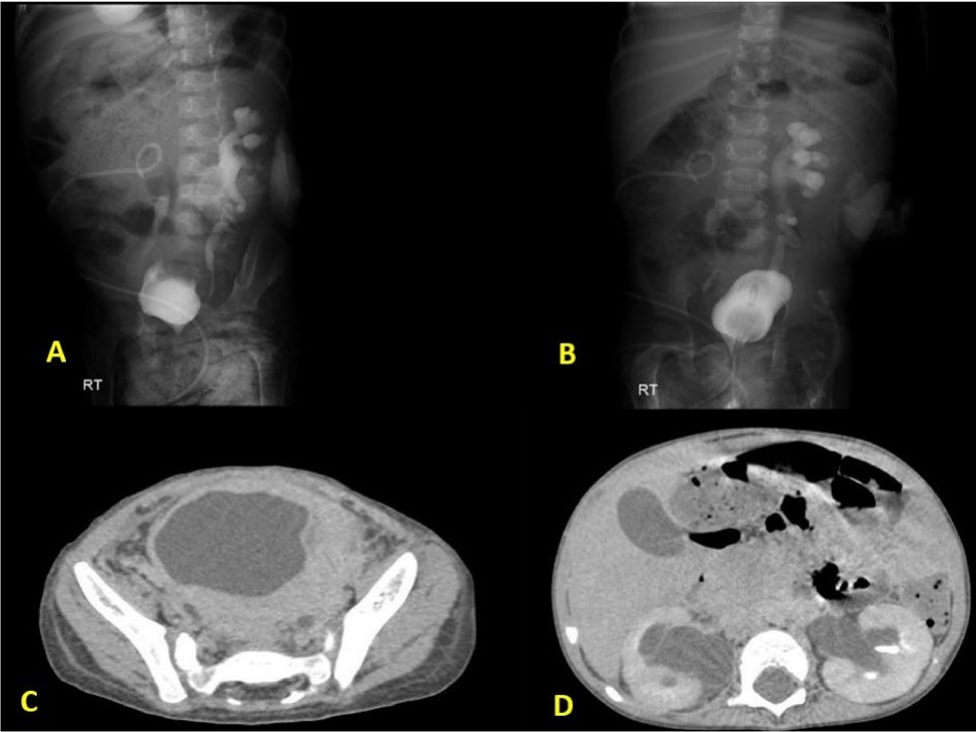
The patient referred with bilateral hydronephrosis, **A** and **B**) bilateral hydronephrosis secondary to involvement of both side of ureterovesical junction by infilterative process. **C**) Wall thickening of the bladder. **D**) moderate pelvicaliatasis

**Fig. 3:**
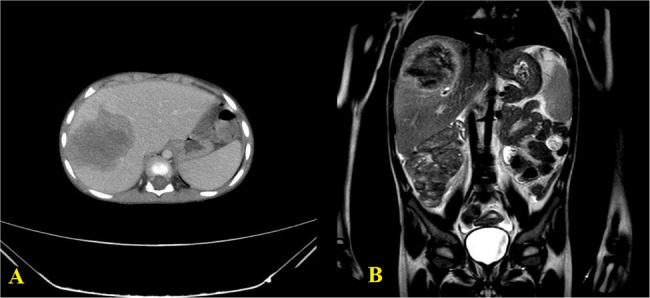
(**A**) Portovenous phase of axial CT scan with contrast shows liver mass. (**B**) Coronal T2W image of the same liver mass

In the next step, these patients’ documents related to medical imaging (CT scan (Fig. 1), radiographs (Fig. 2-A and B), barium studies, ultrasound (Fig. 1-J), and other imaging) were evaluated. All radiological studies were evaluated regarding the lesion location, appearance, size, accompanying involvement (lymphadenopathy), the form of involvement, and visible complications (rupture, obstruction, hydronephrosis (Fig. 2), and other complications (Fig. 3)). It should be noted that a specialized radiology assistant performed all examinations of this study.

The statistical analysis of this study has been changed or clarified in this way: Data were analyzed using SPSS software version 23. Descriptive statistics were employed to summarize demographic findings, including mean, standard deviation, minimum, and maximum for quantitative variables. Categorical variables were presented as frequencies and percentages. Qualitative variables’ relationship was explored using both the chi-squared and Fisher’s exact test. Chi-squared testing was performed for larger sample sizes with appropriate expected frequencies; Fisher’s exact was applied for cases with small sample sizes where the assumptions of chi-squared testing couldn’t be applied appropriately. Quantitative variable distribution was evaluated using Kolmogorov-Smirnov testing. Based on that test: outcomes and the sample size, suitable parametric or non-parametric tests were taken for further analysis. The level of significance was deemed statistically significant at *P* < 0.05. Most of the association symptoms with their initial diagnosis were analyzed by logistic regression focusing on the specific symptoms that correlate with their imaging findings. An effort also extended towards finding independent factors associated with locoregional ileocecal manifestation concerning their demographic background location into outcomes.

## Results

This study examined 32 patients including 17 (53.1%) males and 15 (46.9%) females with a definitive diagnosis of basidioblomycosis with a mean age of 9.84 ± 13.78 with the age range of 1 to 62 years. The population comprised 7 (21.9%) adults (≥ 15 years) 25 (78.1%) pediatric cases (< 15 years) including 13 (40.6%) 0–5 years, 12 (37.5%) 6–15 years old. Demographic information of patients, marital status, habitat, and the prevalence of underlying diseases such as minor thalassemia, diabetes, celiac disease, cystic fibrosis, favism, Hirschsprung, and addiction were assessed and shown in Table 1. The examination of the patients showed that the thickness of the involved bowel wall was 11.40 ± 10.62 mm, and on average, the mass size was 41.87 ± 35.58 mm.

**Table 1:** Patients’ demographic information, marital status, habitat, and the prevalence of underlying diseases, their significance and correlation analysis

Variable	Subgroup	*n* (%)	Context/Significance	Correlation Assessment (Verified)	*p*-value	Test Used
Sex	Male	17 (53.1)	Gender distribution	No correlation with symptom severity	0.41	Fisher’s Exact
	Female	15 (46.9)				
Age Group	Pediatric (< 15 yrs)	25 (78.1)	Predominant pediatric involvement	Thinner wall thickness vs. adults (11.2 ± 4.1 mm)	0.03*	Mann-Whitney U
	Adult (≥ 15 yrs)	7 (21.9)				
Marital Status	Single	29 (90.6)	Demographic characteristic	No clinical correlation	0.87	Chi-square
	Married	3 (9.4)				
Comorbidities	None	25 (78.1)	Most patients immunocompetent	Trend toward better outcomes (NS)	0.15	Fisher’s Exact
	Diabetes	1 (3.1)				
	Minor Thalassemia	2 (6.3)				
	Celiac + Cystic Fibrosis	1 (3.1)				
	Hirschsprung	1 (3.1)				
	Thalassemia + Favism	1 (3.1)				
	Addiction	1 (3.1)				
Habitat	Fars Province	15 (46.9)	Geographic distribution	No correlation with outcomes	0.62	Chi-square
	Southern Provinces (Bushehr and Hormozgan)	17 (53.1)				
Imaging	CT	20 (62.5)	Primary diagnostic modality	Higher diagnostic accuracy vs. MRI	0.008*	Fisher’s Exact
	MRI	5 (15.6)	Detailed assessment			
	CT + MRI	6 (18.8)	Comprehensive evaluation			
	CT + Nephrogram	1 (3.1)	Renal-specific			

Symptoms included fever, abdominal pain, nausea, vomiting, and headache. The initial diagnoses differed from appendicitis, malignancy, hydatid cyst, Inflammatory bowel disease (IBD), Pyonephrosis, Abdominal mass, pancreatitis, and unknown (Table 2). Fever in combination with abdominal pain and vomiting (40.6%) in an individual was likely to be associated with mesenteric masses (OR = 3.2, *p* = 0.03) that would have developed, whereas weight loss would probably be one of the symptoms associated with hepatic lesions (75 vs. 25%, *p* = 0.01). Strong relationship existed between misdiagnosis as malignancy (37.5%) and hepatic/mesenteric masses (83%, *p* < 0.001), however, appendicitis (31.2%) was significantly much more in ileocecal involvement (70%, *p* = 0.02).

**Table 2:** Symptoms and initial diagnosis of patients and statistical analysis

Variable	Subgroup	*n* (%)	Comparison/Correlation	*p*-value	Test Used
Symptoms	Abdominal pain + nausea/vomiting	6 (18.8%)	More common in adults (≥ 15 yrs) vs. pediatric	0.04*	Fisher’s Exact
	Fever + abdominal pain + nausea/vomiting	13 (40.6%)	Associated with mesenteric masses (OR = 3.2, 95% CI:1.1–9.8)	0.03*	Logistic Regression
	Abdominal pain + nausea/vomiting + weight loss	4 (12.5%)	Linked to hepatic lesions (75% vs. 25%, *p* = 0.01)	0.01*	Chi-square
	Fever + abdominal pain + nausea/vomiting + headache	6 (18.8%)	No significant associations	0.22	Fisher’s Exact
	Fever of unknown origin	1 (3.1%)	N/A (single case)	–	–
Initial Diagnosis	Appendicitis	10 (31.2%)	More frequent in ileocecal involvement (70% vs. 30%)	0.02*	Chi-square
	Malignancy	12 (37.5%)	Associated with hepatic/mesenteric masses (83% vs. 17%)	< 0.001*	Fisher’s Exact
	Hydatid cyst	3 (9.4%)	All hepatic lesions (100%, *p* = 0.002)	0.002*	Fisher’s Exact
	IBD	2 (6.3%)	Misdiagnosed in colonic wall thickening cases	0.08	Fisher’s Exact
	Pyonephrosis/Abdominal mass/Pancreatitis/Unknown	5 (15.6%)	No significant patterns	0.45	Chi-square

**Fig. 4:**
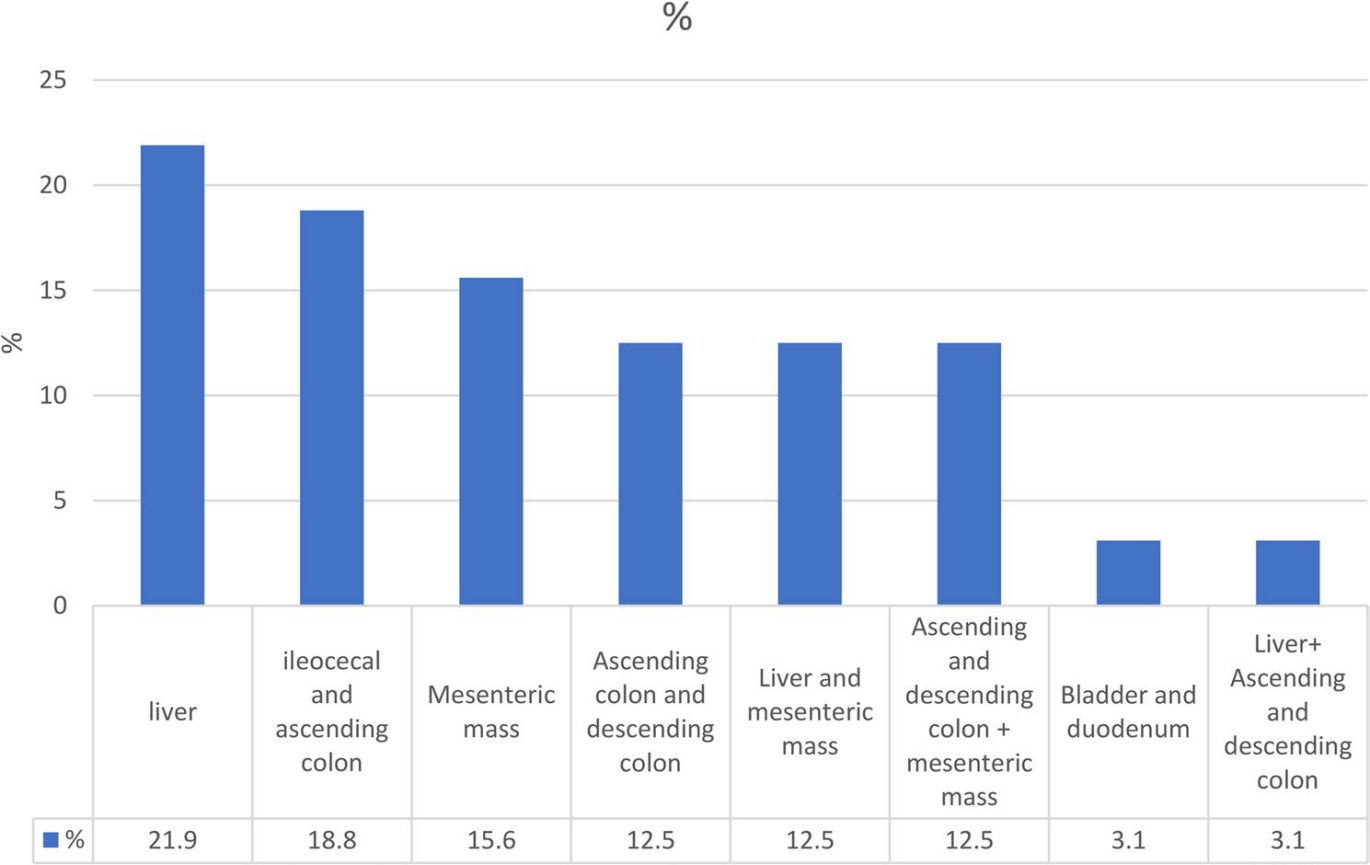
Lesion location in the radiological findings of the study population

**Fig. 5:**
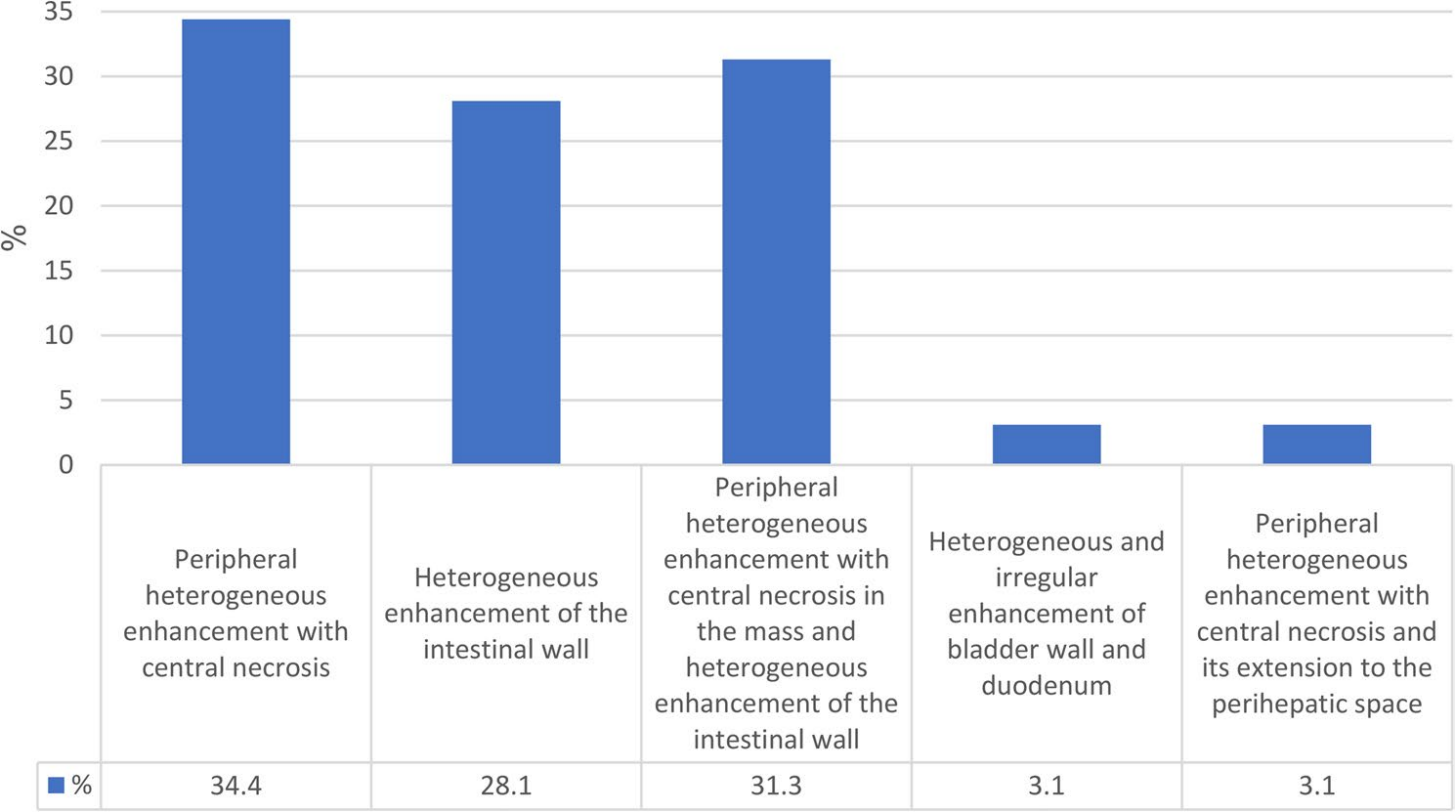
Appearance of lesions in the radiological findings of the studied population

The radiological examination from a sample of cases revealed the finding of hepatic involvement in 21.9% cases (7/32), which demonstrated heterogeneously enhancing masses with central necrosis and were predominantly located in the right lobe (Figs. 1H and 3A-B, and lesion distribution in Fig. 4). Ileocecal and ascending colon lesions (18.8%, 5/32) showed circumferential wall thickening with preserved mucosal layers (Figs. 1F, I, J, frequency of radiological findings in Fig. 5), while mesenteric masses (15.6%, 5/32) presented characteristic peripheral enhancement with necrotic cores (Figs. 1A-E). Enhancement patterns fell into two main categories, Type 1 (34.4%, 11/32) exhibiting peripheral heterogeneous enhancement with central necrosis (as seen in Figs. 1A-E and 3A) and Type 2 (31.3%, 10/32) displaying combined mass necrosis with bowel wall enhancement (on display in Figs. 1F, I). Such different imaging patterns provide key diagnostic features with which basidiobolomycosis can be differentiated from malignancies or inflammatory conditions.

**Fig. 6:**
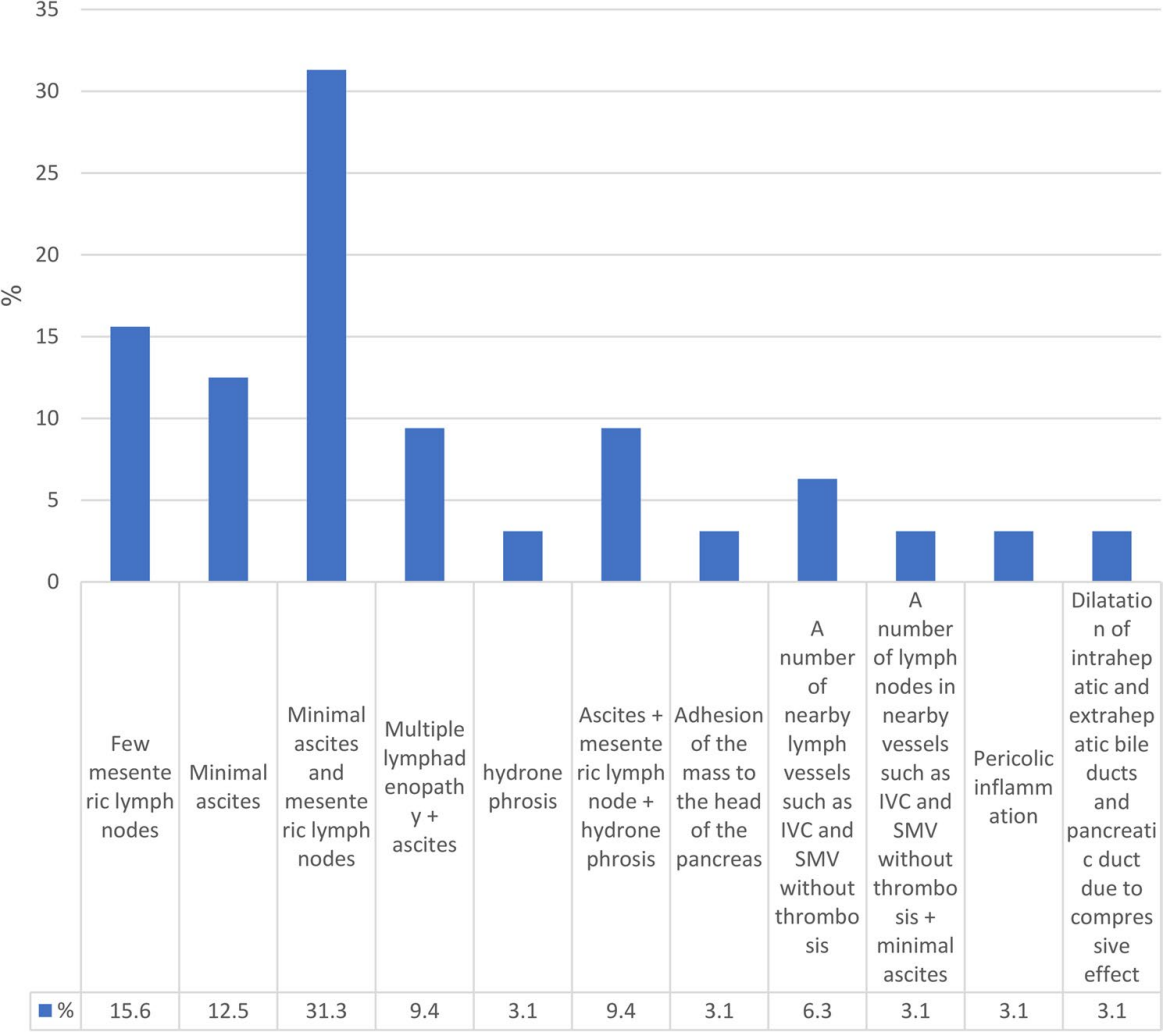
Associated conflicts in radiological findings

Radiological findings showed that 31.3% of involvements with lesions in patients are minimal ascites along with several mesenteric lymph nodes (Fig. 6).

**Fig. 7:**
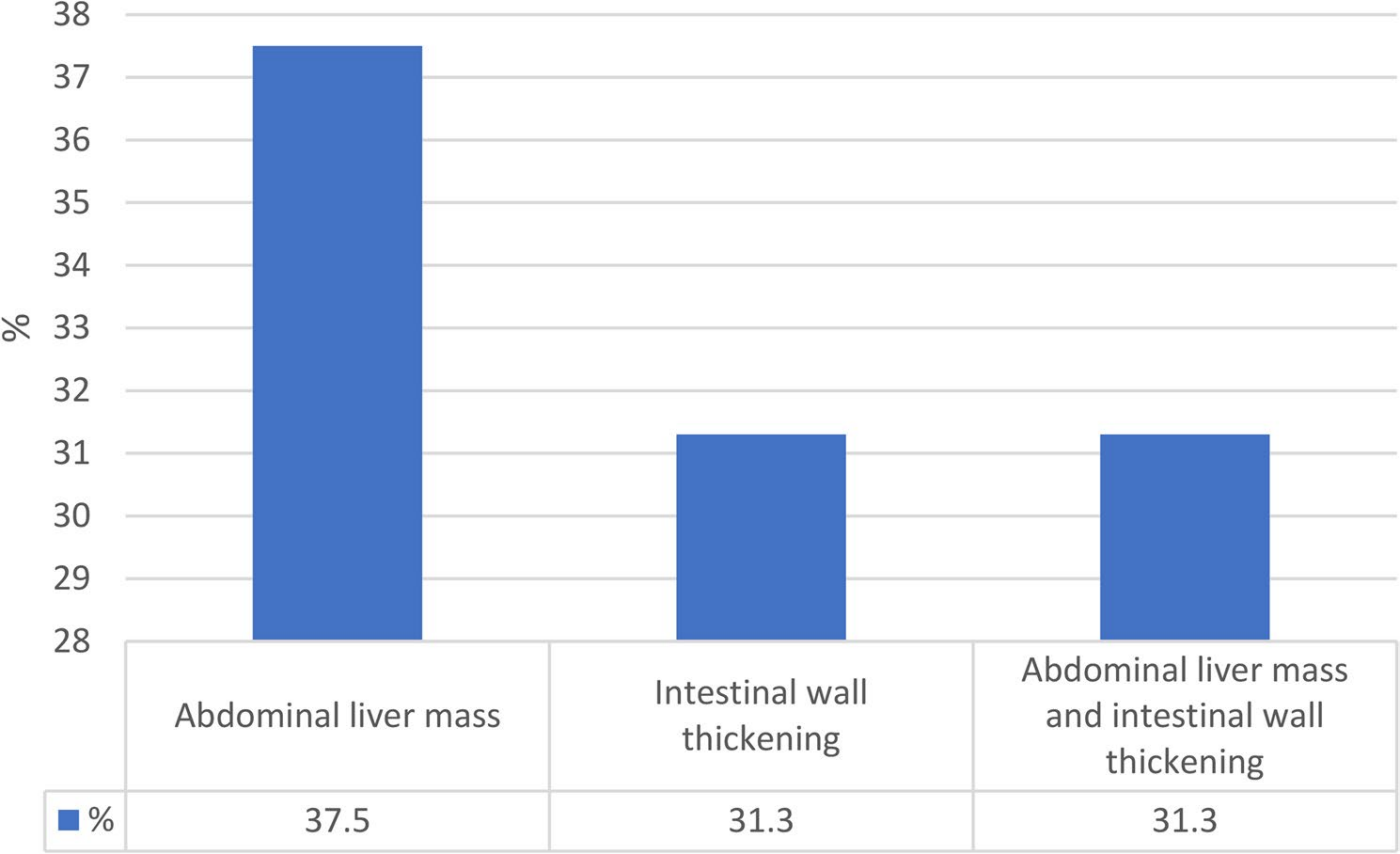
Form of involvement in radiological findings

37.5% of lesions were observed as liver-abdominal mass and 31.3% as an increase in the thickness of the intestinal wall (Fig. 7).

Examinations after the treatment of patients showed that 9.4% of the cases were deceased, and the rest recovered.

Isolated hepatic involvement was noted in 7 out of the 32 cases (21.9%), which occurred only in pediatric patients (age range 2–6 years, mean 3.7 ± 1.7 years). All lesions showed pathognomonic imaging features of peripheral heterogeneous enhancement with internal necrosis (Fig. 3), predominantly in the right lobe (segments 4–7, 85.7%). Mass sizes ranged from 24 × 27 mm to 75 × 67 × 66 mm (mean 47.2 ± 18.1 mm). Larger lesions (> 50 mm) caused significantly frequent vascular displacements, but did not affect overall outcomes (*p* = 0.15). Notably, all of them were misdiagnosed initially - hepatoblastoma/lymphoma (71.4%) or hydatid cyst (28.6%) - concerned with diagnosis. Associated findings: universal lymphadenopathy (max node 14 mm) and ascites in 28.6% of cases, the latter present in sole mortality (3-year-old female with 34 × 26 × 17 mm S6 lesion) (Fig. 3). Findings appropriate to make a case for considering isolated hepatic basidiobolomycosis in this age group under the children with an eosinophilia, especially when associated with peripheral enhancement patterns in right lobe segments.

Extrahepatic involvement associated with hepatic disease was seen in 9 of 32 cases (28.1%) (Table 1) and presented two different patterns of spread: (1) contiguous hepatomesenteric extension (*n* = 5) in which adjacent mesentery is noted to have direct invasion from liver segments 4/5/8 with 80% containing vascular encasement (SMV/PV)(no vascular encasement was seen in isolated liver lesions (*p* < 0.0001 by Fisher’s Exact Test)); and (2) non-contiguous multi-organ dissemination (*n* = 4) in which liver lesions are marked “on but not attached to” distal bowel lesions (mean distance: 12.4 ± 3.7 cm) (Fig. 1A-E shows examples). Such “liver-plus” lesions showed significantly more considerable size in the hepatic mass than pure liver cases (68.3 ± 28.1 mm vs. 47.2 ± 18.1 mm, *p* = 0.03 by Mann-Whitney U test), as well as 100% peripheral necrosis and 88.9% >10 mm lymphnodes. Of liver-plus, adults (≥ 15 years) were 44.4% while for isolated liver lesions, there were no cases at all (*p* = 0.02, by Fisher’s Exact Test). This finding signifies that the spread pattern varies according to age. The most serious case was that of a 33-year-old male with S8 hepatic and 125 mm pelvic mesenteric masses (Fig. 1A), which caused death despite treatment. These findings highlight that hepatic basidiobolomycosis with extrahepatic involvement is a clinically distinct entity characterized by aggressive radiological features and worse outcomes.

Seven out of our population exhibited ileocecal involvement (21.9%) (Table 1), presenting significant circumferential thickening (22.9 ± 6.2 mm; 95% CI: 17.6–28.2; 17–33 mm range) with heterogeneous enhancement. In pediatric patients (6/7), wall thickening was notably less but more diffuse than in the one adult case (20.2 ± 5.1 mm vs. 33 mm, *P* = 0.04). The most prominent associated features were pericolonic fat stranding (85.7%), small reactive lymph nodes (mean size of 12.3 ± 4.7 mm; 8.5–16.3 mm; 95% CI), and ascites (OR 6.25, 95% CI: 1.02–38.3; *P* = 0.04; significant in comparison to 71.4% vs. 28.6% in non-ileocecal cases). On multivariate analysis, age < 15 years (β=−0.51, *p* = 0.02) and absence of liver lesions (β = 0.63; *p* = 0.01) were found to be independent predictors of ileocecal localization (R²=0.72, *p* = 0.003). The medical treatment resulted in 100% resolution despite all cases being initially misdiagnosed as appendicitis, whereas the nonileocecal lesions had only a 12% case fatality rate (*p* = 0.32). Mural stratification was maintained, and obstruction was absent, enabling differentiation of these fungal lesions from Crohn’s disease or malignancy.

## Discussion

The present study investigated the radiological findings of patients with a definite diagnosis of basidioblomycosis in Shiraz from 2015 to 2021. The findings indicated that the number of affected men was more than the number of women in the study population, confirming the findings of previous studies [5, 10, 15].

While Vikram et al. [16] established the epidemiological disease burden on colonic (82%) and hepatic (30%) involvement in their multinational cohort, our radiologic analysis provides extensive measurements previously unreported: hepatic lesions showed a constant peripheral enhancement with central necrosis (Fig. 3), and mesenteric masses greater than 50 mm had a correlation with vascular encasement (*p* < 0.0001). These imaging criteria provide quantifiable measures filling a gap in their clinicopathological model, allowing for objective markers differentiating basidiobolomycosis from malignancies.

The population’s average age was 9.84, which mainly showed that children were involved. A previous study reported the age range of 5 to 10 years. However, this infection can happen at any age [9, 17]. Our results confirmed that extrahepatic lesions were significantly more common in adults (44.4% vs. 0% in isolated hepatic cases, *p* = 0.02), suggesting age-dependent disease progression.

According to the results, most of the patients presented with abdominal pain, nausea, vomiting, and fever. The same as the previous studies reported the symptoms mainly as abdominal pain and fever [3, 5, 16, 18]. Crucially, we found that fever with associated abdominal pain or vomiting was more likely to occur in mesenteric masses (OR = 3.2, *p* = 0.03), while weight loss was associated with hepatic lesions (75% vs. 25%, *p* = 0.01). Such imaging-symptom associations might be useful in prioritizing differential diagnoses.

The results showed that the initial diagnosis for patients was mainly malignancy and appendicitis, which was in line with previous studies; the initial diagnosis was reported mainly as gastrointestinal malignancy or appendicitis [3, 11, 15, 16] which was a wrong diagnosis. Our results revealed that misdiagnosis as malignancy was significantly associated with hepatic/mesenteric masses (83%, *p* < 0.001) and mainly for appendicitis misdiagnosis for ileocecal involvement (70%, *p* = 0.02).

The significance of these radiological findings in clinical practice is considerable. Identification of peripheral enhancement with central necrosis in hepatic or mesenteric lesions should raise the possibility of basidiobolomycosis in endemic areas, which could spare some patients from unnecessary biopsies or surgeries. For thickening of the ileocecal wall, maintained mural stratification (Fig. 1F, I) aids in the differentiation of this process from Crohn’s disease which may help in decision-making under immunosuppression. In addition, vascular encasement in “liver-plus” lesions (80% of contiguous spread cases) demands that attempting resection be preceded by antifungal therapy.

In the present study, most of the population had no severe chronic diseases, only some records of diabetes, minor thalassemia, celiac, cystic fibrosis, Hirschsprung, favism, and addiction. This finding was consistent with the study of Vikram et al., in which most of the patients had no underlying disease, and only a few had diabetes [16]. However, our study has newly determined that localization at ileocecal was predicted independently by age below 15 years (β=−0.51, *p* = 0.02) and by absence of liver lesions (β = 0.63, *p* = 0.01), thus refining anatomical risk stratification.

The location of lesions in the radiological findings of the studied population of this research was mostly in the liver, ileus, and ascending colon. In the study of Vikram et al., most of the areas involved in the lesions were seen in the large intestine, small intestine, and liver [16].

In the present study, the radiological findings of lesion involvement were in the form of a liver-abdominal mass and intestinal wall thickening, the same as in previous studies [3]. This cohort provided a unique observation that mesenteric masses >50 mm (Fig. 1A-E) were associated with vascular encasement (*p* < 0.0001), implying that such would require preoperative antifungal therapy to reduce surgical morbidity.

patients responded to treatment, though three deaths (9.4%) underscored critical risk factors not previously quantified in imaging studies: all had either (a) mesenteric masses >100 mm with vascular envelopments (SMV/PV) or (b) hepatic lesions with associated ascites and hydronephrosis. In contrast, mortality in Vicram et al.‘s [16] cohort correlated with palpable masses (43%), thus suggesting that radiological invasiveness (vascular/ureteral involvement) may be a better predictor of outcome as compared to physical exam findings. It is worth noting that delayed diagnosis (>6 weeks) was universally seen in fatalities (*p* = 0.03), providing prognostic value for the early use of CT/MRI to detect these features.

While our findings provide useful imaging criteria, some points need to be considered as the limitation of the study: First, the retrospective design of the study. Second, although all cases were read independently by two expert radiologists, the absence of inter-observer statistics (e.g., Cohen’s κ) needs to be stated. This replicates clinical practice wherein agreement is often substituted for statistical analysis, but upcoming multicenter trials ought to quantify agreement to enhance protocol generalizability.

## Conclusion

This study defines unique radiological patterns in the appearance of basidiobolomycosis radically improving the distinction with common abdominal pathologies. With key imaging hallmarks being peripheral enhancement and central necrosis in hepatic lesions, together with preserved mural stratification in intestinal involvement, these imaging characteristics become essential diagnostic tools for our study cohort. This implies significant management considerations, since understanding these patterns can prevent unnecessary interventions and peripheral enhancement with central necrosis should prompt immediate antifungal initiation, while preserved mural stratification may obviate surgery.

The suggestions reveal that specific radiological features are liable to an association with clinical outcomes because there are certain high-risk features identifiable with poorer prognosis. This imaging risk-stratification model offers an argument to apply diagnostic imaging tools in time and for the best treatment in an early setting for patients from endemic areas. The consistent presentation of these patterns seems to urge for a protocol that includes imaging for making an early diagnosis and consequently averting a delay in outcomes.

Having a retrospective study design, these results make a real advance beyond the description of a rare syndrome; they empirically describe a series of imaging criteria. Prospective research could add a different edge to this, such as validation against molecular diagnostics and algorithmic assessment on antifungal management. For now, radiologists ought to include these specific findings in their thought processes on abdominal masses in these vulnerable areas, even in the face of a less-than-clear clinical picture.

## Data Availability

Data is provided within the manuscript or supplementary information files.Written informed consent was obtained from all adult participants, as well as from the parents or legal guardians of children under 16.
